# Determinants for early introduction of complementary foods in Australian infants: findings from the HSHK birth cohort study

**DOI:** 10.1186/s12937-020-0528-1

**Published:** 2020-02-18

**Authors:** Amit Arora, Narendar Manohar, Debra Hector, Sameer Bhole, Andrew Hayen, John Eastwood, Jane Anne Scott

**Affiliations:** 1 School of Health Sciences, Western Sydney University Campbelltown Campus, Locked Bag 1797, Penrith, NSW 2751 Australia; 2 Translational Health Research Institute, Western Sydney University, Locked Bag 1797, Penrith, NSW 2751 Australia; 3 Discipline of Child and Adolescent Health, Sydney Medical School, Faculty of Medicine and Health, The University of Sydney, Westmead, NSW 2145 Australia; 4 Oral Health Services, Sydney Local Health District and Sydney Dental Hospital, NSW Health, Surry Hills, NSW 2010 Australia; 5 Cancer Australia, Surry Hills, NSW 2010 Australia; 6 Sydney Dental School, Faculty of Medicine and Health, The University of Sydney, Surry Hills, NSW 2010 Australia; 7 Oral Health Alliance, Oral Health Centre, University of Queensland, Brisbane, QLD 4006 Australia; 8 Metro North Oral Health Services, Stafford, QLD Australia; 9 Australian Centre for Public and Population Health Research, Faculty of Health, University of Technology Sydney, Ultimo, NSW Australia; 10 Community Paediatrics, Sydney Local Health District, NSW Health, Croydon, Australia; 11 School of Women’s and Children’s Health, University of New South Wales, Kensington, NSW Australia; 12 School of Medicine, Griffith University, Gold Coast, QLD Australia; 13 School of Public Health, Curtin University, Perth, WA Australia

**Keywords:** Complementary feeding, Solids, Infants, Cohort study, Australia

## Abstract

**Objective:**

The purpose of this study was to examine the timing of introduction of complementary (solid) foods among infants in South Western Sydney, Australia, and describe the maternal and infant characteristics associated with very early introduction of solids.

**Methods:**

Mother-infant dyads (*n* = 1035) were recruited into the “Healthy Smiles Healthy Kids” study by Child and Family Health Nurses at the first post-natal home visit. Data collected via telephone interviews at 8, 17, 34 and 52 weeks postpartum included timing of introduction of solids and a variety of maternal and infant characteristics (*n* = 934). Multiple logistic regression was used to identify factors independently associated with the risk of introducing solids very early, which for the purpose of this study was defined as being before 17 weeks.

**Results:**

The median age of introduction of solids was 22 weeks. In total, 13.6% (*n* = 127) of infants had received solids before 17 weeks and 76.9% (*n* = 719) before 26 weeks of age. The practice of introducing solids early decreased with older age of the mother. Compared to women < 25 years of age, those who were 35 years or older were 72% less likely to introduce solids very early (OR = 0.28, CI_95_ 0.14–0.58). Single mothers had more than twice the odds of introducing solids before the age of 17 weeks compared to married women (OR = 2.35, CI_95_ 1.33–4.16). Women who had returned to work between 6 to 12 months postpartum were 46% less likely to introduce solids very early compared with those who were not working at the child’s first birthday (OR = 0.54, CI_95_ 0.30–0.97). Women born in Vietnam and Indian sub-continent had lower odds of introducing solids very early compared to Australian born women (OR = 0.42, CI_95_ 0.21–0.84 and OR = 0.30, CI_95_ 0.12–0.79, respectively). Infants who were exclusively formula-fed at 4 weeks postpartum had more than twice the odds of receiving solids very early (OR = 2.34, CI_95_ 1.49–3.66).

**Conclusions:**

Women who are younger, single mothers, those not working by the time of child’s first birthday, those born in Australia, and those who exclusively formula-feed their babies at 4 weeks postpartum should be targeted for health promotion programs that aim to delay the introduction of solids in infants to the recommended time.

## Background

The process of gradual introduction of complementary foods into an infant’s diet is essential for meeting the nutritional needs of infants in their first year of life [1]. The decision around when to start introducing complementary foods to their infant is a dilemma faced by every mother. Complementary foods represent all liquid, semi-solid, and solid foods other than breast milk, infant formulas and follow-on formulas [2] either commercial or home-made [3]. It is advised that when semi-solid and solid complementary foods (hereafter referred to as solids) are introduced to an infant, their textures should be changed as appropriate to the age of the infant so as to give a variety of textural experiences [4].

From a paediatric health perspective, the timing of introducing solids is a sensitive issue due to the potential effects on children’s long-term health status [5, 6]. Presently, the World Health Organization (WHO) recommends infants should be exclusively breastfed until the age of 6 months, followed by the introduction of nutritious solids to complement on-going breastfeeding [7]. Such a feeding pattern ensures optimal growth and positive health benefits [8]. This guideline has been supported in a slightly modified form by the Australian National Health and Medical Research Council (NHMRC) [9] and the American Academy of Pediatrics [10], with both organisations recommending that solids should be introduced ‘around’ or ‘at about’ 6 months of age. However, certain international organisations’ guidelines slightly differ. For example, the European Society of Paediatric Gastroenterology Hepatology and Nutrition (ESPGHAN) [11, 12] and European Food Safety Authority [13] recommend that complementary foods be introduced “no earlier than 17 weeks and no later than 26 weeks”. Further, the Australian recommendations were recently updated by consensus at an Infant Feeding Summit in May 2016 and it is currently recommended that “When your infant is ready, at around 6 months, but not before 4 months, start to introduce a variety of solids, starting with iron rich foods, while continuing breastfeeding [14].

Undertaking research on complementary feeding practices is essential to identify specific population sub-groups of women who decide to introduce solids early and the reasons for not complying with international recommendations. A variety of factors that influence early introduction of solids have been reported in the literature [15], however these factors vary across regions, populations, cultures, and countries [16–18]. In Australia, recent studies report that many parents introduce solids at early ages [19, 20]. National statistics from the Australian Institute of Health and Welfare show that around 35% of four-month old’s had consumed soft/semi-solid foods [19]. The New South Wales (NSW) Child Health Survey (2009–2010) reported that 44.6% of infants were introduced solids before 6 months of age [21].

While national and state-wide statistics are available which indicate the prevalence and predictors of early introduction of solids in infants, there are limited data for families residing in South Western Sydney (SWS) region of NSW. The SWS is a part of Greater Western Sydney region and is considered to be one of the most culturally diverse and socially-disadvantaged populations in Australia [22–24].

The purpose of the current study was three-fold:

- to examine the timing of introduction of solids to infants residing in South Western Sydney;
- to ascertain the sociodemographic and biomedical predictors associated with very early introduction of solids in this population group; and
- to investigate the association of the timing of introduction of solids with the duration of breastfeeding.

These data will indicate the degree of accordance with the existing Australian and International infant feeding recommendations. Additionally, they will assist in identifying women most at risk of introducing solids early and targeting of interventions to improve infant feeding practices with respect to the timing of introduction of solids among particular sub-populations in Australia.

## Methods

### Study background

This study analyses data collected as part of the ongoing Healthy Smiles Healthy Kids (HSHK) cohort study in South Western Sydney that began in late 2009 and which has been described previously [22]. In brief, women who gave birth to a live infant with no serious health condition between October 2009 and February 2010 in public hospitals located under the catchment of the former Sydney South West Area Health Service (now classified as Sydney and South Western Sydney Local Health Districts) were approached to be a part of this study. Child and Family Health Nurses (CFHNs) recruited mother-infant dyads at the first post-natal home visit at four to 6 weeks, as this is the primary point of community-based health professional contact for newborn children and their parents/carers [25]. At the first post-natal visit, CFHNs explained the project to the mothers and obtained written informed consent. If requested, the nurses were able to arrange for interpreter services for non-English speaking parents/carers and language-appropriate written materials were provided for the major ethnic groups (such as Vietnamese, Chinese, Indian sub-continent and Arabic) living in this region.

### Data collection

Basic demographic, biomedical and infant feeding information were collected via a baseline telephone interview conducted when the child was 8 weeks old. Follow-up interviews were conducted at 17, 34 and 52 weeks postpartum. The questionnaire used in this study was adapted from the first and second Perth Infant Feeding studies [16, 17, 26]. At each interview, information was collected on infant feeding practices including breastfeeding, the use of infant formula, and the introduction of complementary foods including solids and other fluids. If mothers had introduced solids to their infants, they were asked a closed-ended question on the primary reason for early introduction of solids to their infants.

### Outcome measure

The outcome measure for this study was the age (in weeks) at which solids were introduced for the first time in infants. Very early introduction of solids was considered to be before 17 weeks of age, considering the recommendations from the 2016 Australian Infant Feeding Summit [14] and the ESPGHAN [11, 12].

### Exposure measures

A variety of sociodemographic and biomedical characteristics identified in other studies and considered to be associated with the age of introduction of solids were investigated. The sociodemographic variables included maternal age, mother’s education level, marital status, mother’s and her partner’s country of birth, mother’s occupation at 12 months postpartum, mother’s employment status at 12 months postpartum, and socioeconomic status. Mothers’ provided their residential postcode and this information was used to classify their socioeconomic status (SES) as per the Census Index of Relative Socioeconomic Disadvantage. The biomedical factors include gestational age, parity, infant’s gender, infant birth weight, delivery method, initiation and duration of breastfeeding, feeding method at 4 weeks postpartum, and maternal smoking and alcohol intake during or after pregnancy.

### Statistical analysis

The Statistical Package for Social Sciences, Version 24 (SPSS for Windows, SPSS Inc., Chicago, IL, USA) was used to analyse the data. The primary reason for introducing solids to the infants before 17 weeks were analysed using frequency distribution.

Univariate logistic regression was initially employed to explore the relation between introduction of solids before 17 weeks and each individual explanatory variable. Later, a multivariate logistic regression analysis was performed to determine which variables were independently predictive of the introduction of solids before 17 weeks of age. All explanatory variables were entered into the full model which was reduced using the backward stepwise procedure (p for removal < 0.05) and the fitness of model was assessed at every step to avoid dropping non-significant variables that affected the model fitness. All variables in the final model were variables for which, when excluded, the change in deviance compared with the corresponding *Χ*^2^ test statistic on the relevant degrees of freedom was significant.

Survival analysis was used to examine the association between timing of introduction of solids with the duration of breastfeeding. The effect of timing of introduction of solids on the duration of breastfeeding was evaluated using the Kaplan-Meier estimate of “survival” (continuation of breastfeeding) and the log-rank test was used to assess the quality of the survival curves.

### Ethical considerations

Ethics approvals for this study were obtained from the former Sydney South West Area Health Service – RPAH Zone (ID number X08–0115), Liverpool Hospital, University of Sydney, and Western Sydney University. All participants signed a written consent form to be a part of this study.

## Results

**Fig. 1:**
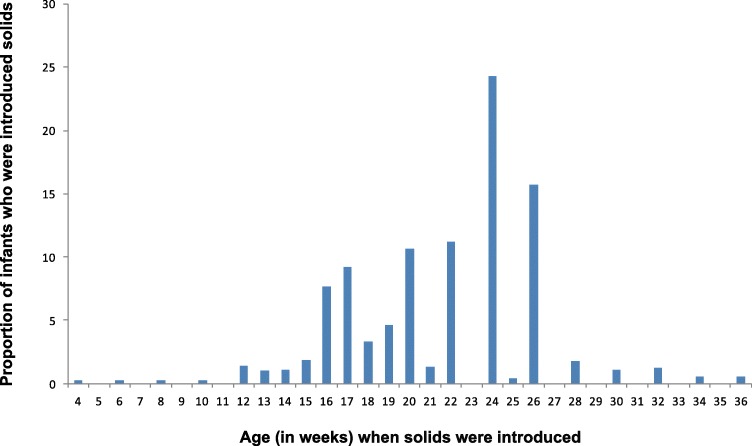
The distribution of age at which solid foods were first introduced

Of the 1035 mother-infant dyads that were recruited into the HSHK study, 934 completed the interviews at 8, 17, 34, and 52 weeks. The median age for the introduction of solids was 22 weeks (Interquartile range 18, 24) with the peak timing of introduction of solids at 24 weeks (Fig. 1). In total, 13.6% (*n* = 127) of infants had received solids before 17 weeks and 76.9% (*n* = 719) received their first solids before 26 weeks of age.

**Fig. 2:**
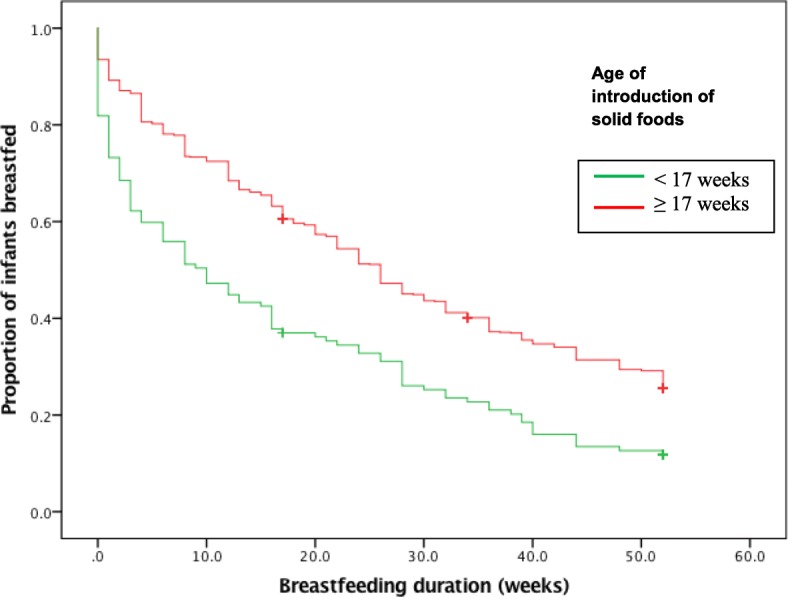
The association of breastfeeding duration and age of introduction of solid foods

There was a significant association between the timing of introduction of solids and the duration of breastfeeding (log rank test *X*^*2*^ = 31.71, df = 1, *p* < 0.001) (Fig. 2). The median breastfeeding duration for mothers who introduced solids at or after 17 weeks breastfed was 27.6 weeks compared with 17.5 weeks for those mothers who introduced solids before 17 weeks.

A variety of sociodemographic factors were associated with very early introduction of solids (Table 1). Single women were more likely than married women to introduce solids to their babies very early. Whereas older women, University-educated women, and women in professional occupations were less likely to introduce solids before 17 weeks compared to women who were younger, dropped out of school, or listed their occupations as home duties or student, respectively. Women who migrated to Australian from Vietnam, China, the Indian sub-continent and other Asian countries were less likely to introduce solids before 17 weeks compared to those born in Australia.

**Table 1:** Association between sociodemographic factors and very early introduction of solid foods (< 17 weeks) (*n* = 934)

Variable	Solids introduced^a^	Solids introduced^a^	Univariate odds ratio^b^	
	< 17 weeks	≥17 weeks		
	N (%)	N (%)	OR	CI_95_
Maternal age (years)				
< 25	30 (32.6)	62 (67.4)	1.00	
25–29	36 (14.1)	219 (85.9)	0.34***	0.19, 0.60
30–34	44 (12.5)	307 (87.5)	0.30***	0.17, 0.51
≥ 35	17 (7.2)	219 (92.8)	0.16***	0.08, 0.31
Maternal education				
< Year 12	33 (23.4)	108 (76.6)	1.00	
Year 12 completed	36 (17.3)	172 (82.7)	0.69	0.40, 1.16
College/TAFE	23 (12.9)	155 (87.1)	0.49*	0.27, 0.87
University	35 (8.6)	372 (91.4)	0.31***	0.18, 0.52
Marital Status				
Married	79 (10.8)	655 (89.2)	1.00	
Living with a partner/ De facto	18 (16.4)	92 (83.6)	1.62	0.93, 2.83
Single Mother	30 (33.3)	60 (66.7)	4.15***	2.52, 6.81
Mother’s country of birth				
Australia	77 (17.7)	359 (82.3)	1.00	
China	3 (5.4)	53 (94.6)	0.26*	0.80, 0.87
Vietnam	12 (9.1)	120 (90.9)	0.47*	0.25, 0.89
Asia Other	6 (5.5)	104 (94.5)	0.27**	0.11, 0.64
Middle-East/ Africa	9 (11.1)	72 (88.9)	0.58	0.28, 1.22
Others	20 (16.9)	98 (83.1)	0.95	0.55, 1.63
Partner’s country of birth				
Australia	59 (16.4)	300 (83.6)	1.00	
China	2 (4.8)	40 (95.2)	0.25	0.06, 1.08
Vietnam	8 (7.0)	107 (93.0)	0.38*	0.18, 0.82
Asia Other	7 (6.7)	98 (93.3)	0.36*	0.16, 0.82
Middle-East/ Africa	12 (12.5)	84 (87.5)	0.73	0.37, 1.41
Others	12 (10.0)	108 (90.0)	0.57	0.29, 1.09
Mother’s occupation				
Home duties/ student	32 (18.8)	138 (81.2)	1.00	
Unskilled	22 (13.0)	147 (87.0)	0.65	0.36, 1.17
Sales/Clerical	43 (14.7)	250 (85.3)	0.74	0.45, 1.23
Managers	9 (14.8)	52 (85.2)	0.75	0.33, 1.67
Professionals	21 (8.7)	220 (91.3)	0.41**	0.23, 0.74
Mother’s employment status				
Not working at 12 months	71 (14.5)	420 (85.5)	1.00	
Return to work < 6 months	33 (17.6)	155 (82.4)	1.26	0.80, 1.98
Return to work 6–12 months	18 (8.1)	203 (91.9)	0.53	0.31, 0.90
Index for Relative Socioeconomic Disadvantage				
Least Disadvantaged	6 (12.0)	44 (88.0)	1.00	
2nd Quintile	36 (15.1)	202 (84.9)	1.31	0.52, 3.29
3rd Quintile	7 (8.8)	73 (91.3)	0.70	0.22, 2.23
4th Quintile	19 (10.4)	164 (89.6)	0.85	0.32, 2.25
Most Disadvantaged	59 (15.4)	324 (84.6)	1.34	0.55, 3.28

^a^ The total of the categories do not always add up to 934 due to missing or incomplete data for some items

^b^ The univariate odds ratio indicates the likelihood of early introduction of solid foods

*OR* odds ratio, CI_95−_95% confidence interval

* *p* < 0.05 ** *p* < 0.01 *** *p* < 0.001

The list of biomedical factors associated with very early introduction of solids are shown in Table 2. Mothers who had initiated breastfeeding were less likely to introduce solids before 17 weeks compared with those who did not initiate breastfeeding. Whereas those women who were formula-feeding at 4 weeks postpartum and those who smoked cigarettes during pregnancy or were current smokers were more likely to introduce solids to their babies very early.

**Table 2:** Association between biomedical factors and very early introduction of solid foods (< 17 weeks) (*n* = 934)

Variables	Solids introduced^a^	Solids introduced^a^	Univariate odds ratio^b^	
	< 17 weeks	≥17 weeks		
	N (%)	N (%)	OR	CI_95_
Parity				
Primiparous	61 (13.1)	406 (86.9)	1.00	
Multiparous	66 (14.1)	401 (85.9)	1.09	0.75, 1.59
Infant gender				
Female	59 (12.9)	398 (87.1)	1.00	
Male	68 (14.3)	409 (85.7)	1.12	0.77, 1.63
Infant birth weight				
≥ 2500 g	121 (13.6)	767 (86.4)	1.00	
< 2500 g	6 (13.0	40 (87.0)	0.95	0.39, 2.29
Gestational age				
≥ 37 weeks	120 (13.9)	746 (86.1)	1.00	
< 37 weeks	7 (10.3)	61 (89.7)	0.71	0.32, 1.60
Method of delivery				
Vaginal	94 (14.4)	558 (85.6)	1.00	
Caesarean	33 (11.7)	249 (88.3)	0.79	0.52, 1.20
Breastfeeding initiation				
No	23 (30.7)	52 (69.3)	1.00	
Yes	104 (12.1)	755 (87.9)	0.31***	0.18, 0.53
Feeding method at 4 weeks				
Fully breastfed	62 (10.6)	524 (89.4)	1.00	
Partially breastfed	13 (9.0)	131 (91.0)	0.84	0.45, 1.57
Fully formula fed	52 (25.5)	152 (74.5)	2.89***	1.92, 4.36
Smoking status of the mother postpartum				
No	100 (11.8)	751 (88.2)	1.00	
Yes	27 (32.5)	56 (67.5)	3.62***	2.19, 6.00
Smoking status of the mother during pregnancy				
No	108 (12.2)	775 (87.8)	1.00	
Yes	19 (37.3)	32 (62.7)	4.26***	2.33,7.78
Alcohol drinking status of the mother postpartum				
No	78 (12.3)	554 (87.7)	1.00	
Yes	49 (16.2)	253 (83.8)	1.38	0.93, 2.03
Alcohol drinking status of the mother during pregnancy				
No	111 (13.2)	731 (86.8)	1.00	
Yes	16 (17.4)	76 (82.6)	1.39	0.78, 2.46

^a^ The total of the categories do not always add up to 934 due to missing or incomplete data for some items

^b^ The univariate odds ratio indicates the likelihood of early introduction of solid foods

*OR* odds ratio; CI_95_–95% confidence interval

*** *p* < 0.001

Table 3 shows the factors that independently predict the very early introduction of solids. After adjusting for covariates, single mothers had more than twice the odds of introducing solids before the age of 17 weeks compared to married women (OR = 2.35, CI_95_ 1.33–4.16). The odds of introducing solids very early decreased with increasing maternal age and compared to women less than 25 years of age, those who were 35 years or older were 72% less likely to introduce solids very early (OR = 0.28, CI_95_ 0.14–0.58). Women who had returned to work between 6 to 12 months postpartum were 46% less likely to introduce solids very early compared with those who were not working at 12 months postpartum (OR = 0.54, CI_95_ 0.30–0.97). Compared to women born in Australia, migrant women from Vietnam (OR = 0.42, CI_95_ 0.21–0.84) and other Asian countries other than China (OR = 0.30, CI_95_ 0.12–0.79) were less likely to introduce solids to their infants before 17 weeks.

**Table 3:** Sociodemographic and biomedical factors independently^a^ associated with very early introduction of solid foods (< 17 weeks) (*n* = 934)

Variable^b^	Mean age of introduction of solids (weeks)	AdjOR^c^	CI_95_	*p*-value
*Sociodemographic factors*				
Maternal age (years)				
< 25	19.65	1.00		
25–29	21.52	0.53	0.28, 0.99	0.048
30–34	21.87	0.47	0.26, 0.87	0.015
≥ 35	22.26	0.28	0.14, 0.58	0.001
Mother’s country of birth				
Australia	20.97	1.00		
China	22.58	0.39	0.12, 1.32	0.131
Vietnam	23.44	0.42	0.21, 0.84	0.013
Asia Other	23.22	0.30	0.12, 0.79	0.014
Middle-East/ Africa	21.51	0.64	0.29, 1.39	0.261
Others	20.51	1.07	0.60, 1.93	0.815
Maternal employment status				
Not working at 12 months post-partum	21.77	1.00		
Returned to work < 6 months post-partum	21.20	1.42	0.87, 1.92	0.160
Returned to work 6–12 months post-partum	21.83	0.54	0.30, 0.97	0.039
Marital Status				
Married	21.98	1.00		
Living with a partner/ De facto	21.59	1.19	0.65, 2.18	0.577
Single Mother	19.12	2.35	1.33, 4.16	0.003
*Biomedical factors*				
Feeding method at 4 weeks				
Fully breastfed	21.97	1.00		
Partially breastfed	21.94	0.98	0.51, 1.92	0.970
Fully formula fed	20.58	2.34	1.49, 3.66	0.000

^a^ Non-significant variables were partner’s country of birth, mother’s occupation, index of relative socioeconomic disadvantage, parity, infant gender, infant birth weight, mother took antibiotics during pregnancy and labour, smoking status of the mother during pregnancy, alcohol status of the mother in pregnancy and postpartum, method of delivery

^b^ All variables in the final model were variables for which, when excluded, the change in deviance compared with the corresponding χ^2^ test statistic on the relevant degrees of freedom was significant

^c^*AdjOR* Adjusted odds ratio, CI_95−_95% confidence interval

Only one biomedical factor was independently associated with the risk of introducing solids very early: mothers who were exclusively formula-feeding their infants at 4

s postpartum were more than twice as likely to introduce solids very early (OR = 2.34, CI_95_ 1.49–3.66) compared to those who were fully breastfeeding at 4 weeks postpartum.

Table 4 shows the mothers’ self-reported reasons for very early introduction of solids (*n* = 127). The main reasons given were: their baby was hungry (*n* = 45, 35.4%), their baby was old enough to start solids (*n* = 33, 26.0%), they were advised by family and/or peers (*n* = 21, 16.5%), they used solids to settle the baby or help them to sleep through the night (*n* = 15, 11.8%) and/or they believed their baby was showing an interest in solids (*n* = 13, 10.3%), for example by putting their hands or other objects in their mouth and chewing on them or showing an interest in the parent’s food.

**Table 4:** Reasons for introducing solid foods before 17 weeks of age (*n* = 127)

Reason	N	%
Baby hungry	45	35.4
Baby old enough to wean	33	26.0
Advised by family and/or friends	21	16.5
To settle the baby/help him/her sleep at night	15	11.8
Baby interested^a^	13	10.3

^a^ Interest indicated by baby putting hands and other objects into mouth and/or chewing hands and other objects or interest towards the parent’s food

## Discussion

Since 2003, Australian mothers have been recommended to introduce solids to their infants at around 6 months of age [9, 27]. This ongoing cohort study commenced in late 2009, when this recommendation had been in place for approximately 6 years. However, a recent consensus from the Australian Infant Feeding Summit emphasised that “When your infant is ready, at around six months, but not before four months, start to introduce a variety of solids, starting with iron rich foods, while continuing breastfeeding” [14]. Similarly, ESPGHAN [11, 12] and European Food Safety Authority [13] recommend that complementary foods should be introduced “no earlier than 17 weeks and no later than 26 weeks”. While nearly 80% of infants in this study were introduced solids before 26 weeks, only 13.6% had received solids very early (before 17 weeks or 4 months). This percentage is much lower than that observed in the Perth Infant Feeding Study II (PIFS-II) [16] conducted in 2002/2003 when the recommended timing of introduction of solids was ‘between 4 and 6 months’. In PIFS-II, 44% of infants had received solids before 17 weeks and 93% before 6 months. Median age of introduction of solids in the current study was 22 weeks which was almost 4.5 weeks later than in the PIFS-II [16].

The 2010 Australian National Infant Feeding Survey [28] reported that 28.4% infants residing in NSW and 35.3% infants nationally within Australia received soft/semi-solid/solid foods by the age of 4 months. Hence, the current study showed better concordance with the infant feeding recommendations. Overall, the results of the present study and other contemporaneous Australian studies [28, 29] suggest a gradual shift towards introduction of solids closer to the recommended timing at the population level [9].

In this current study, a significant negative association was reported between very early introduction of solids and the duration of breastfeeding. Those mothers who introduced solids at or after 17 weeks breastfed their children an average of 10 weeks longer than those who introduced solids before 17 weeks. This finding is consistent with studies from France [30], England [31], Denmark [32] and other studies in Australia [16]; all of which found that early introduction of solids was associated with a shorter duration of breastfeeding.

There was an independent association between very early introduction of solids and certain sociodemographic and biomedical factors. Younger mothers were more likely to introduce solids very early, which is also a common finding in previous studies [29–31]. Several studies have recognised single mothers as a potential predictor for shorter duration of breastfeeding and early introduction to solids [15, 33, 34]. This association was also seen in the current study. It has been suggested that this association is due to increased stress from a lack of paternal support [33].

In the current study, employment status of the mother was defined as ‘time lapse for returning to work following child birth’ i.e., if and when a mother had returned to work during the first 12 months postpartum. The association between employment status and age of introduction was not in the direction expected and mothers who resumed work within 6–12 months after giving birth were less likely to introduce solids very early compared to mothers who had not returned to work at 12 months postpartum. While, returning to work within 6 months post-partum compared to not returning to work at 12 months was not associated with very early introduction of solids. Other studies have found no association between early introduction of solids and employment status [16, 30].

Maternal ethnicity (country of birth) was found to be a strong predictor for very early introduction of solids with mothers born in Vietnam and other Asian countries including the Indian sub-continent, were less likely to introduce solids very early to their infants compared with mothers born in Australia. This suggests that very early introduction of solids might be influenced by some cultural and ethnic factors. Ethnic and cultural associations of early introduction of solids have been reported in the literature [16, 35, 36]. An earlier study on infant feeding practices in Sydney found that Vietnamese-born women had optimal infant feeding practices as a result of remaining in a close community network maintaining traditional customs [37]. Similarly, having support from family and health professionals from a similar cultural background may support optimal infant feeding practices [38]. In contrast, a recent systematic review examined complementary feeding practices of South Asian women living in the United Kingdom (UK) and in South Asian countries (Manikam et al., 2016). Among women who had migrated to the UK, there was lower accordance with recommended infant feeding practices than among women who remained in their country of birth and these practices were influenced by low acculturation levels and conflicting information received from health professional, family elders, and community leaders. In this study, reasons for not introducing solids earlier by Vietnamese and Indian mothers were not explored and it is therefore difficult to draw conclusions.

Mothers who fully formula-fed their infant at 4 weeks postpartum were twice as likely to introduce solids earlier than 17 weeks compared to mothers who were fully breastfeeding their infants at 4 weeks postpartum. This finding aligns with a previous Australian study [39]. In a study in China, Tang et al. [18] reported that infants given formula regularly within the first 6-months of life were at a higher risk of receiving complementary foods early. Exclusive formula feeding is believed to be linked with impairment of appetite self-regulatory mechanisms which leads to infants demanding solids earlier without subsequent reduction in milk consumption during the complementary feeding phase [40]. Such impairment in early stage of life might pose long-term health complication such as increased risk of overweight and obesity in adulthood [41].

Among mothers that introduced solids to their babies before 17 weeks, the foremost self-reported reason was that they perceived that their child was “hungry” and breast milk and/or formula alone could not satisfy their child’s appetite. Brown and Rowan [42], reported ‘infant hunger’ as the principal reason for early introduction of solids, with other reasons being ‘infant weight and behaviour’. Similar findings have been reported in other studies [4, 43]. ‘Pressure and/or advice from others’ was also a commonly reported reason for early introduction of solids in these studies [4, 42, 43], and this was also observed in current study. In the current study, mothers also perceived that their babies were ‘ready for solids’. Similar finding has been reported elsewhere and it is considered that multiple sources of information such as health practitioners, family, friends, and media can be conflicting and insensitive to the needs of mothers [44]. Effective interventions are therefore needed to educate mothers on the scientifically recommended age window for introducing solids rather than relying on their personal judgement on infant developmental readiness.

### Study strengths and limitations

Mothers from socioeconomically disadvantaged and ethnically diverse groups, which are often under-represented in research of this kind, were the focus of this study. The data were collected prospectively soon after birth and at three additional time points over a total 12 month period postpartum, thereby minimising the potential of ‘recall bias’ and ‘heaping of data’ [45] in relation to events of interest. The time of introducing solids was measured in weeks rather than months which allows for precise measurement of the time of event (i.e., the age of introducing solids) and clearly described early introduction of solids as “before 17 weeks”. Many studies [34, 46] report infant feeding patterns in months and define early introduction of solids as “before 4 months” and it therefore remains unclear if this refers to completed months of age. Researchers also do not provide a standardised criterion for converting months to weeks or vice versa and these conversions are often inconsistently determined and reported. Such differences make it difficult to compare findings across studies. It is highly recommended that “17 weeks of age” and “26 weeks of age” to be consistently adopted as the definition for four and 6 months of age, respectively.

There are several limitations of this study. First, the participants were recruited from public hospitals located in South Western Sydney therefore the observed associations in the current study may not reflect associations at the populations level within New South Wales or Australia at the time of this study. Second, the outcomes were measured based on self-reporting which might have led to social desirability bias. Further, for certain explanatory variables e.g., country of birth, the number of women in respective categories was small (< 5). Since a relatively small proportion of women introduced solid foods early, this resulted in a rare events bias which was reflected as large confidence intervals around the odds ratio [47]. Hence, a larger study sample of women would have provided more statistically robust findings and current study findings should be interpreted with care. Also, the peak timing of introduction of solids was at 24 weeks which might suggest that many women would have interpreted 24 weeks to be 6 months (based on the assumption that 4 weeks is equal to 1 month).

## Conclusion

In a sample of 934 mother infant dyads in South West Sydney, the median age of introduction of solids was 22 weeks. Nearly 80% of mothers had introduced solids by the time their infant was 26 weeks (6 months) of age and 14% of mothers had introduced solids to their infants very early (before 17 weeks). Mothers who were young, single, and fully formula-feeding their infants at 4 weeks of age were more likely to introduce solids very early. Mothers born in Australia were also more likely to introduce solids very early. Mothers at risk of introducing solids very early should be provided broader social support to enable them to make informed decisions regarding the timing of introducing solids to their infants. Additionally, existing infant feeding guidelines and promotion initiatives should incorporate specific sections on educating mothers on how to interpret infant behaviour and what needs to be done if their child seems hungry and unsettled.

## Data Availability

The data of this study can’t be shared publically due to the presence of sensitive (confidential) participants’ information.
